# Tocotrienol-Adjuvanted Dendritic Cells Inhibit Tumor Growth and Metastasis: A Murine Model of Breast Cancer

**DOI:** 10.1371/journal.pone.0074753

**Published:** 2013-09-19

**Authors:** Sitti Rahma Abdul Hafid, Srikumar Chakravarthi, Kalanithi Nesaretnam, Ammu Kutty Radhakrishnan

**Affiliations:** 1 Pathology Division, Faculty of Medicine and Health, International Medical University, Bukit Jalil, Kuala Lumpur, Malaysia; 2 Nutrition Unit, Malaysian Palm Oil Board, Bandar Baru Bangi, Selangor, Malaysia; Faculty of Medicine, University of Porto, Portugal

## Abstract

Tocotrienol-rich fraction (TRF) from palm oil is reported to possess anti-cancer and immune-enhancing effects. In this study, TRF supplementation was used as an adjuvant to enhance the anti-cancer effects of dendritic cells (DC)-based cancer vaccine in a syngeneic mouse model of breast cancer. Female BALB/c mice were inoculated with 4T1 cells in mammary pad to induce tumor. When the tumor was palpable, the mice in the experimental groups were injected subcutaneously with DC-pulsed with tumor lysate (TL) from 4T1 cells (DC+TL) once a week for three weeks and fed daily with 1 mg TRF or vehicle. Control mice received unpulsed DC and were fed with vehicle. The combined therapy of using DC+TL injections and TRF supplementation (DC+TL+TRF) inhibited (p<0.05) tumor growth and metastasis. Splenocytes from the DC+TL+TRF group cultured with mitomycin-C (MMC)-treated 4T1 cells produced higher (p<0.05) levels of IFN-γ and IL-12. The cytotoxic T-lymphocyte (CTL) assay also showed enhanced tumor-specific killing (p<0.05) by CD8^+^ T-lymphocytes isolated from mice in the DC+TL+TRF group. This study shows that TRF has the potential to be used as an adjuvant to enhance effectiveness of DC-based vaccines.

## Introduction

Dendritic cells (DCs) are highly specialized antigen presenting cells (APC) that play a key role in mediating anti-tumor immune responses [1,2]. These cells possess unique properties that can elicit primary and boost secondary immune responses as well as to regulate the type of immune responses mediated by T-cells [2,3]. In recent years, several studies have demonstrated that tumor antigen-pulsed DC cells, otherwise known as cancer vaccines, are capable of inducing activation and proliferation of both T-helper (TH) cells and cytotoxic T-lymphocytes (CTL) to mediate anti-tumor immune responses [4,5]. A number of studies have evaluated the therapeutic potential of DC-based cancer vaccines for some tumors such as breast, lung, colon and prostate cancers. However, in many of these studies, the efficacy of the DC-based vaccines against established tumors in mice and humans appears to be low [6-11]. Hence, there is a need to improve the efficacy of these cancer vaccines in established tumors, possibly through the use of adjuvants.

Tocotrienols and tocopherols are fat-soluble vitamins that belong to the vitamin E family. Tocopherols are found most abundantly in oil extracted from soybean, cottonseed and sunflower seed whilst tocotrienols are found primarily in palm oil, cereal grains such as wheat, barley and rice. Tocotrienol-rich fraction (TRF) is a natural compound isolated from palm oil, which has been shown to be non-toxic to normal cells [12,13,14]. The TRF contains about 70% tocotrienols and 30% of alpha-tocopherol [15]. Tocotrienols have been the focus of increasing research interest in the last five to ten years as a unique nutritional compound. There is growing scientific evidence, which show that tocotrienols have many health-promoting effects such as antioxidants [16], cholesterol-lowering effects [17], cardio-protective effects [18] as well as anti-cancer properties [12,13,19-21].

We have previously found that the combined approach of vaccinating mice with DC-pulsed with tumor lysate (DC+TL) from the 4T1 cells and daily supplementation with TRF prior to the induction of tumor markedly inhibited tumor growth and produced tumor-specific response [11]. This study suggested that TRF can be used as an adjuvant-therapy to boost the immune-enhancing and anti-cancer effects of DC-based cancer vaccines. Although the findings from this study suggested that this approach has good potential to be developed as an immunotherapeutic approach, its clinical application was limited as cancer patients only sought for treatment once the cancer has been established and to date, we do not have a definite tool to identify individuals who may develop cancer. Hence, in the present study, we evaluated the efficacy of the combination therapy using DC-pulsed with tumor lysate and daily TRF supplementation in mice that already have palpable tumors. The aim of this study was to investigate the effectiveness of supplementation of TRF as an adjuvant to enhance the efficacy of DC-based cancer vaccines to inhibit tumor growth and metastasis in a syngeneic mouse model of breast cancer.

## Materials and Methods

### Mice

Inbred female BALB/c mice (six-week-old) were purchased from the Institute for Medical Research (IMR), Kuala Lumpur, Malaysia and housed at the Animal Maintenance Facility of the same institute. The animals were maintained on commercial pellet diet and water *ad libitum*. The bedding was changed every three-to-four days*.*

### Ethics Statement

All animal work was conducted according to relevant national and international guidelines. All experiments using animals were approved by the Ethics Committee of the Institute of Medical Research, Malaysia. All procedures performed on the animals were in accordance with the approved guidelines.

### Cell line

Murine 4T1 cell line, a spontaneously metastatic tumor cells derived from mammary gland tumor of BALB/c mice was purchased from the American Type Culture Collection (ATCC, Rockville, USA). The 4T1 cells are comparable to human stage IV breast cancer [21]. These cells are poorly immunogenic and express surface MHC class I but not MHC class II molecules. The tumor cells were cultured in 25 ml/cell culture flasks (Nunc, Denmark) as recommended by the ATCC.

### Medium and Cytokines

Complete medium (CM) consisted of RPMI 1640 supplemented with 10% heat-inactivated fetal bovine serum (FBS), 2 mM glutamine and 100 U/mL penicillin-streptomycin solution. For the generation of DC from murine bone marrow (BM), the CM was supplemented with recombinant mouse cytokines such as granulocyte-macrophage colony-stimulating factor (GM-CSF), interleukin-4 (IL-4) and tumor necrosis factor-alpha (TNF-α). All the recombinant mouse cytokines were purchased from Chemicon (USA).

### Supplements

The TRF was obtained as a kind gift from the Golden Hope Plantation Berhad, Malaysia, whilst the Soy oil (Mazola) was purchased from a commercial source.

### Generation of Bone Marrow-derived DC

Murine BM cells were harvested by flushing the marrow cavities of femur and tibia bones of five-to-eight weeks-old BALB/c mice with medium under aseptic condition. Erythrocyte-depleted mouse bone marrow cells were cultured in CM supplemented with GM-CSF (10 ng/ml) and IL-4 (10 ng/ml) at 37°C in a humidified 5% CO_2_ incubator (Heraeus, Germany). The medium was changed every three to four days. On day six, TNF-α (20ng/ml) was added to the DC cultures to induce maturation. The DC was harvested between day seven to nine. The changes in cell morphology was monitored using inverted microscopy (Carl Zeiss, Germany) as described previously [11]. The CD11c expression in the maturing DC was analyzed after seven to nine days of culture using the FACS Calibur flow cytometer (BD Biosciences, California, USA).

### Flow cytometry analyses

The DC generated from murine bone marrow were subjected to direct immunostaining using cell surface-specific marker conjugated monoclonal antibodies and analyzed using FACS Calibur flow cytometer (Becton Dickinson, California, USA) with the Cell-Quest software (Becton Dickinson, California, USA). For the *in-vitro* study, the TRF-treated and untreated DC were stained with relevant fluorochrome-conjugated antibodies to murine cell surface antigens such as CD40, CD80, CD83 and CD86 (Becton Dickinson, California, USA). These molecules are important surface marker on the antigen presenting cells as they provide co-stimulatory signal required for T-cell activation and survival [22,23]. For the *in-vivo* study, leucocytes from the mice from the various experimental groups were harvested from heparinized blood collected from these animals at autopsy and stained with fluorochrome-conjugated antibodies to murine CD40, CD80, CD83 and CD 86 (Becton Dickinson, California, USA). For each antibody, a minimum of three mice per group (n=3) were used. The cells were gated using the forward (FSC) and side (SSC) scatter parameters. For each sample 20,000 cells were collected for analysis.

### Preparation of tumor lysate from 4T1 cells

The 4T1 cells were cultured in the presence or absence of 8 µg/ml TRF in T25 culture flasks overnight. This TRF concentration (8 µg/ml) was chosen (Golden Hope Plantation Berhad, Malaysia) based on our previous work [11,12]. The TRF-treated confluent 4T1 cells were harvested in a 15 ml tube (Falcon, USA). The cells were resuspended in 1 ml complete medium. Tumor lysate (TL) was prepared by subjecting these cells five freeze-thaw cycles where the cells were sequentially frozen in liquid nitrogen and thawed at 65°C for five times. The cell lysates were centrifuged at 2000 rpm for 5 min and passed through a 30 µm nylon filter-column. The lysate was aliquoted and stored at -80°C until use.

### Tumor lysate pulsed DC

The DC was resuspended in a T25 culture flask using fresh medium. Tumor lysate was added into the flask where the ratio of DC: tumor cells used for preparing the lysate was set at 3:1, as previously described [11] and incubated at 37°C for 24 hours in a humidified 5% CO_2_ incubator. Following this, the cells were washed thrice in Hank’s Balance Salt Solution (HBSS) and the DC were recovered through centrifugation (2000 rpm for 3 min). Then, the DC pulsed with tumor lysate (DC + TL) were aliquoted in 1 ml complete medium and injected into the experimental mice. Control DC that was not pulsed with tumor lysate was used as control (DC).

### Induction of tumor in mice

Six-week-old female BALB/c mice were orthotopically injected with 10^4^ 4T1 cells into their mammary gland. After 14 days, the tumor was palpable in all the experimental mice. Then, the animals received an intra-venous injection of DC or DC+TL [1.5 x 10^6^ DC in 50 µl of phosphate-buffered saline (PBS)] once a week for three consecutive weeks. In addition to the DC injections, the mice in the DC+TL+TRF group were also daily supplemented with 1 mg TRF whilst mice in the DC+TL (control) group were fed daily with commercially available Soy oil, which served as the vehicle for the vitamin E supplement. The perpendicular diameters referred to length (L) and width (W) were measured [11] every 2 to 3 days using a digital caliper. The tumor volume (V) was calculated using the following formula: **V = 0.52 × L^2^× W**, which was previously described [12]. Each group consisted of six mice (n=6 per group). The animals were monitored daily to minimize suffering of the animals. The animals were monitored and humanely euthanized when they start to show signs of severe decline in health conditions, distressed or become moribund. The animals were euthanized by placing them in a tank with CO_2_ briefly followed by cervical dislocation. The body weights of the mice were recorded every week and mean tumor volumes were calculated and graph plotted for each group.

### Lung nodule count

At autopsy, the lungs of all the mice used in this study were removed. The images showing the gross morphology of the lungs were captured digitally and the number of tumor nodules on the lungs were counted and recorded in all specimens. The analysis was done using Kruskal-Wallis Test for global comparison of organ metastases among the groups [24].

### Hematoxylin and eosin staining

Once animals were sacrificed, the tumors, lungs and livers were removed and stored in 10% formalin for 48 hours before they were processed for histopathological studies. The tumors and organs were placed in cassettes and placed in automated tissue processor (Leica TP1020 Automatic Tissue Processor, Leica, Germany). The samples were embedded in molten wax and 5 µM thick sections cut using a rotary microtome (Leica, Germany) once the samples were ready. The samples were stained with eosin and hematoxylin (H&E) stain and coded. The coded slides were read by a pathologist, who was blinded. For each group, sections from a minimum of three mice (n=3) were examined for histopathological changes. The analysis was done using Kruskal-Wallis Test for global comparison of organ metastases among the groups [24].

### Mitomycin-C treated 4T1 cells

Confluent 4T1 cells were treated with 10 µg/ml mitomycin-C (MMC) (Sigma, USA) for 2 hours at 37°C in humidified 5% CO_2_incubator. At the end of treatment, the 4T1 cells were washed with PBS and recovered by centrifugation (1,200 rpm x 5 min). The wash step was repeated twice. After the final wash, the MMC-treated 4T1 cells were resuspended in CM.

### Tumor-specific lymphocyte proliferation

At autopsy, the spleen of all the animals was aseptically removed for the tumor-specific lymphocyte proliferation assays. A splenocyte suspension was prepared from each spleen. The cells were seeded into a 96-well culture plate at 5x10^4^ cells/well in triplicates. These cells were co-cultured with equal number of MMC-treated 4T1 cells at 37°C for 72 hours a humidified 5% CO_2_ incubator. After 72 hours, the culture supernatant was collected and the amount of IFN-γ and IL-12 produced by tumor-specific lymphocytes was quantified using commercial ELISA kits as recommended by the manufacturer (eBiosciences, USA). For this assay, spleen from three animals (n=3) per group was used.

### Cytotoxic T-lymphocyte assay

Spleen cells from experimental mice were removed and CD8^+^ T-cells were isolated aseptically using a MACS Microbeads cell separation kit as recommended by the manufacturer (Miltenyi Biotech GmbH, Germany). In the cytotoxic T-lymphocyte (CTL) assay, the CD8^+^ cells isolated from splenocytes serve as effector cells whilst the MMC-treated 4T1 cells served as target cells. The CTL was performed using different effector-to-target (E: T) cell ratios (5:1, 10:1, 15:1 and 20:1) in 96-well round-bottomed plates. The number of target cells was maintained at 1 X 10^4^ cells/ well and the final reaction volume used was 200 µl/ well. The plate was incubated at 37°C for 10 hours a humidified 5% CO_2_ incubator. Following this, 50 µl of the culture supernatant was collected from each well to quantify cytolysis. Cytolysis was determined by quantitatively measuring a commercial lactate dehydrogenase kit according to the manufacturer’s protocol (Promega, Madison, WI, USA). The percentage of cell lysis was determined using the following formulae: {100 x [(experimental release- effector spontaneous release- target effector spontaneous release) / (target maximum release-target spontaneous release)]}. Spleens from three mice per group were used for this assay and for each animal, triplicate sample were used.

### Statistical analysis

The statistical analysis of the tumor growth data of control and treated experimental groups were analyzed using multivariate ANOVA (MANOVA) together with the Tukey HSD post-hoc test. The same test was used for the expressions of CD40, CD80 in cultured cells, expression of cell surface molecules by flow-cytometry in DC and peripheral blood leucocytes (PBL) as well as production of cytokines (IFN-γ and IL-12) and the tumor-specific cytotoxic response by CD8^+^ T-lymphocytes for each E:T ratio. The results are expressed as mean ± SEM. One-way ANOVA test was used to analyze lung nodules in experimental mice. All statistical procedures were performed at 95% confidence level using the SPSS software version 20.0.

## Results

### Antitumor effect by tumor lysate-pulsed DC with TRF supplementation

**Figure 1:**
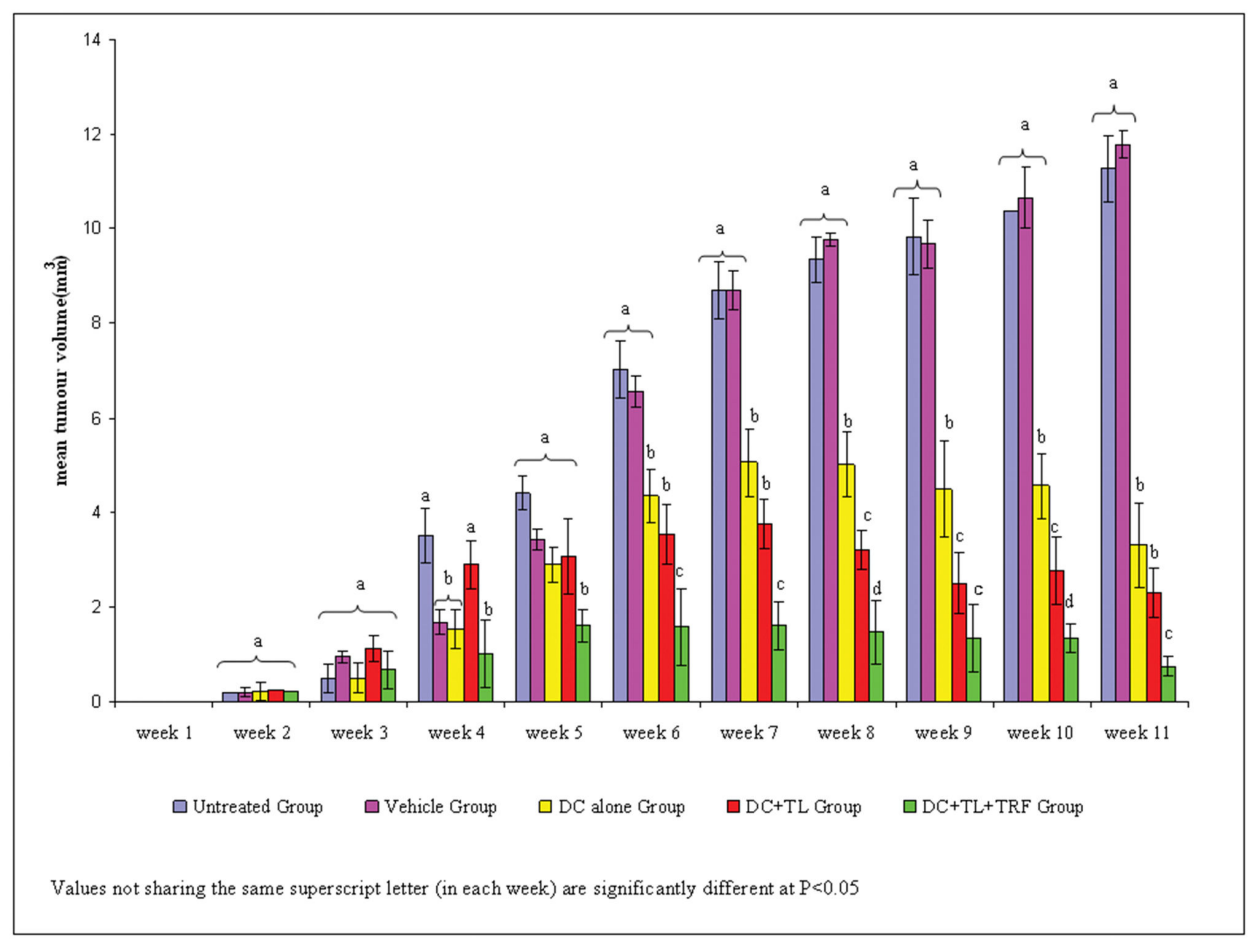
Mean tumor volume + SEM of animals induced with tumor using 4T1 murine mammary cancer cells. Tumor volume was measured every three days from the time tumor was palpable (day 14) until the animals were sacrificed (week 11). Multivariate analysis showed that the DC+TL+TRF treatment was more effective (P < 0.05) than the DC+TL in inhibiting tumor growth. In addition, DC+TL treatment was found to be better than DC alone. [**Untreated**: induced with tumor; **Vehicle**: induced with tumor and fed with Soy oil; **DC**: induced with tumor and treated with DC; **DC+TL**: induced with tumor and treated with DC+TL; **DC+TL+TRF**: induced with tumor and treated with DC+TL and supplemented daily with TRF].

The DC treatment was given once a week for three consecutive weeks once the tumor was palpable (Day 14). As shown in Figure 1, there was a statistically significant difference in tumor growth based on weeks calculated {F(10, 8) = 8.473, p<0.003; Wilks’Λ= 0.086, partial η^2^ = 0.91}. The multiple comparisons between weeks in each group showed that week 7 onward significantly reduced tumor volumes when compared to untreated group. As shown in Figure 1, there was a significant (p<0.05) reduction in tumor growth in the mice from the DC+TL+TRF group. The tumor growth in the mice from this group remained static after the third DC treatment. The mice in the DC+TL and DC alone were fed with 50 µl of Soy oil (vehicle) daily. All the mice in these two groups developed tumor rapidly. The mice in the vehicle supplemented group also developed tumor, which progressively increased in size after week 4. The tumor growth and size in the mice in the DC or DC+TL groups were found to be slower and smaller. At the end of the study (Week 11), the mean tumor volume of the untreated group, vehicle-only, DC alone, DC+TL and DC+TL+TRF were found to be 11.26 ± 2.11 mm^3^, 11.77 ± 0.30 mm^3^, 3.3 ± 0.89 mm^3^, 2.3 ± 1.00 mm^3^ and 0.75 ± 1.20 mm^3^ (mean ± SD) respectively. The result showed that the treatment approach of DC+TL+TRF was the most effective (p<0.05) in controlling tumor growth.

### Metastasis in lungs of experimental mice

**Figure 2:**
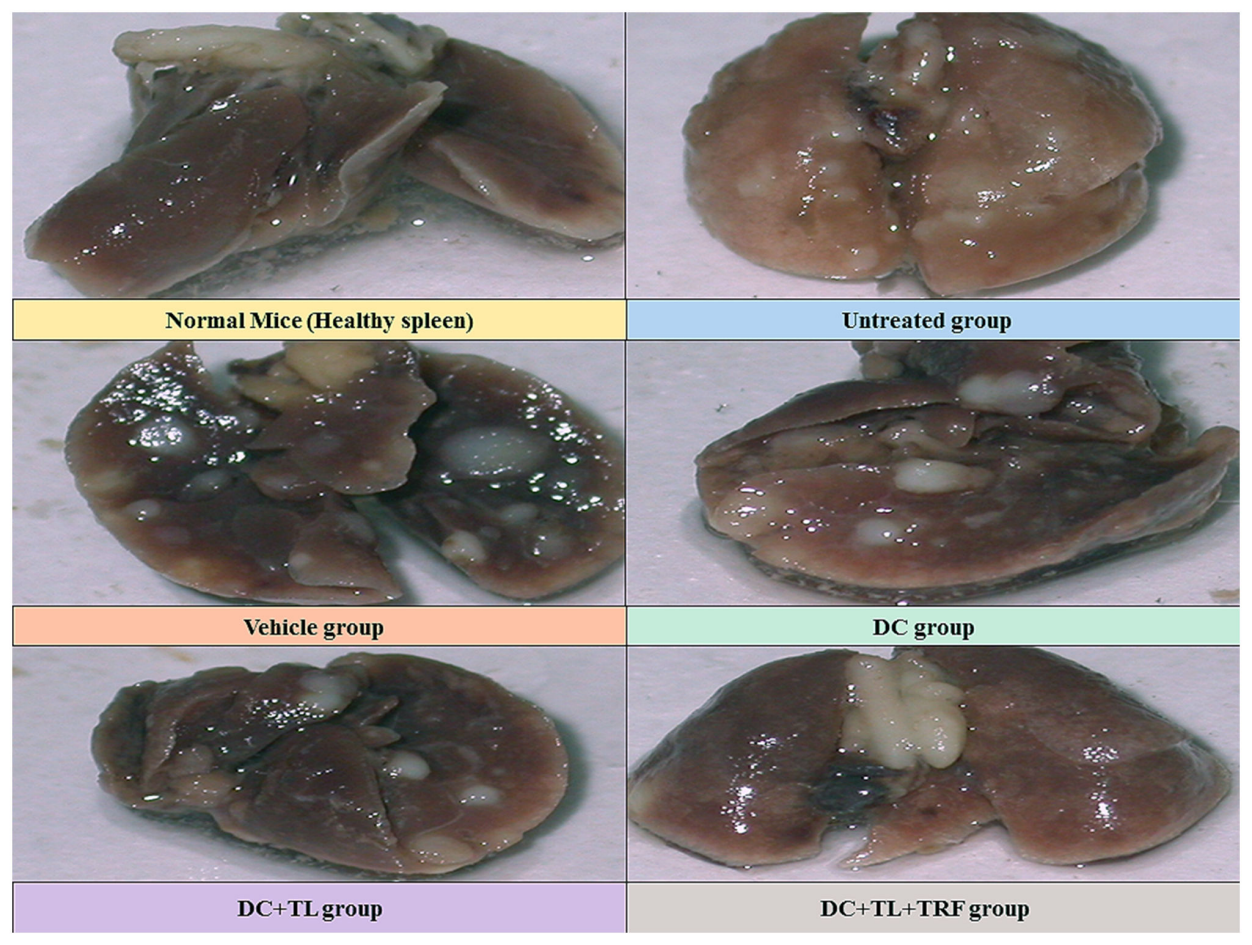
Gross morphology of lungs showing metastatic nodules. Pictures were taken at autopsy once the lungs were removed from the respective animals. [**Normal**: no tumor or treatment; **Untreated**: induced with tumor; **Vehicle**: induced with tumor and fed with Soy oil; **DC**: induced with tumor and treated with DC; **DC+TL**: induced with tumor and treated with DC+TL; **DC+TL+TRF**: induced with tumor and treated with DC+TL and supplemented daily with TRF].

Metastatic tumor nodules that were observed in the lungs of mice from the various groups are shown in Figure 2. There were many nodules observed on the lung excised from the untreated group fed with vehicle, whilst the number of nodules appears to be slightly decreased in the DC group. In contrast, there were very few nodules in the lung from the animals in the DC+TL group whilst in the lung from the DC+TL+TRF group had the least metastasis. The number of nodules observed in the lungs was counted for four mice (n=4) in each group (Table 1). In the untreated group, there was an average of 31.0 ± 1.73 nodules whilst mice in the vehicle group had 22.5 ± 1.44 nodules. There appear to be a significant (p<0.05) reduction of nodules in the lungs taken from mice from the DC (8.0 ± 0.577 nodules), DC+TL (2.0 ± 1.15 nodules) and DC+TL+TRF (0.25 ± 0.25) groups.

**Table 1:** Lung nodule counts.

**Group**	**Nodule count**	**P value < 0.05**	**P value < 0.05**
	(mean + SEM)	(compared to untreated group)	(compared to DC+TL+ TRF)
Normal mice group	0.0000	0.000*	1.000
Untreated group	31.0 ± 1.73	1.000	0.000*
Vehicle group	22.5 ± 1.44	0.000*	0.000*
DC alone group	8.0 ± 0.577	0.000*	0.001*
DC+TL group	2.0 ± 1.15	0.000*	0.849
DC+TL+TRF group	0.25 ± 0.25	0.000*	1.000

^*^ Significant different (p< 0.05)

### Histopathological Grading of Primary Tumor and Organ Metastases

**Figure 3:**
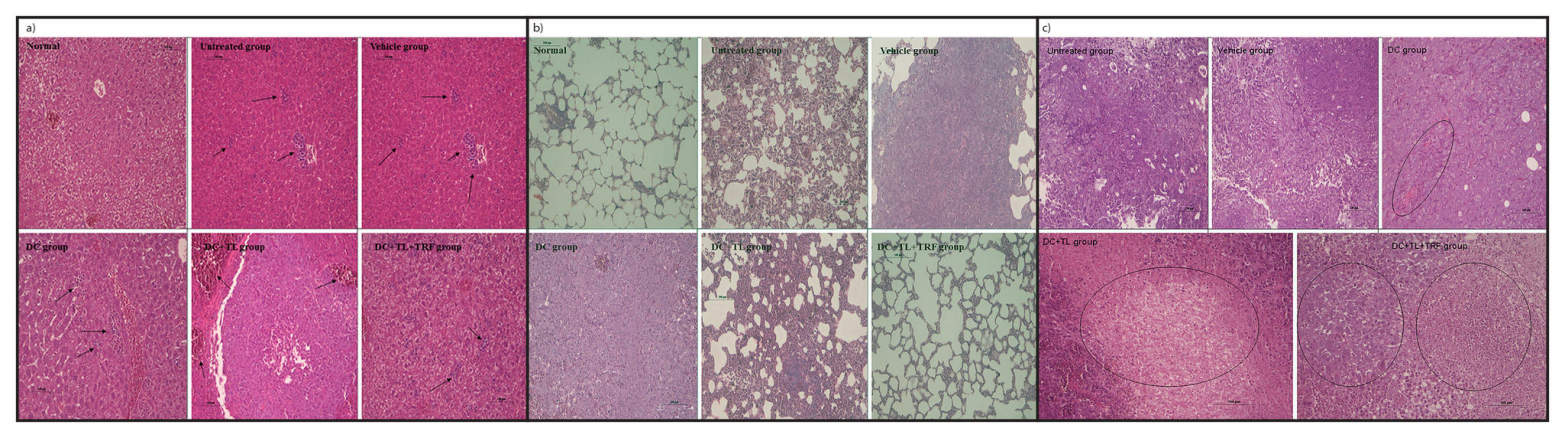
Histopathological changes in the (a) liver {*arrows refer to metastatic deposits in the liver*} (b) lungs and (c) tumor {*circles refer to areas of necrosis*} excised from the animals at autopsy. The figure shows representative of microphotographs of the respective tissue sections stained with the H&E stain (200X magnification). [**Normal**: no tumor or treatment; **Untreated**: induced with tumor; **Vehicle**: induced with tumor and fed with Soy oil; **DC**: induced with tumor and treated with DC; **DC+TL**: induced with tumor and treated with DC+TL; **DC+TL+TRF**: induced with tumor and treated with DC+TL and supplemented daily with TRF].

Table 2 shows the differentiation of the primary breast tumor as well as lung and liver metastases in each group analyzed using the Kruskal-Wallis test for global comparison of organ metastases among group that was previously described [24]. From the test conducted, we found the *p*-value to be 0.005 (*p*<0.05) for the lungs and liver. This means that there is a global difference which is statistically significant among the groups for these organs. The primary tumor was graded accordingly as well differentiated, moderately differentiated or poorly differentiated. **Untreated group**: Presence of a large tumor composed of round to oval cells with large prominent vesicular nuclei and few areas of hyperchromatic nuclei present (Figure 3c). There are few mitotic cells seen. The cells exhibit extensive pleomorphism and are arranged in sheets and clusters, with no evidence of any capsule. Sections from the lung show presence of clusters of metastatic deposits amidst the alveolar regions (Figure 3b). These clusters are numerous and irregular, composed of the same nature of cells seen in the primary tumor. The sections from the liver show normal hepatic architecture with no evidence of metastasis (Figure 3a). **Vehicle group**: Sections from the liver show presence of clusters of metastatic deposits amidst the hepatocytes and around the sinusoids (Figure 3a). The cells exhibit extensive pleomorphism and are arranged in sheets and clusters, with no evidence of any capsule (Figure 3b). The primary tumor is of the same nature as untreated group, composed of round to oval cells with large prominent vesicular nuclei and few areas of hyperchromatic nuclei present (Figure 3c). These clusters are numerous and irregular and contain few to numerous cells. **DC group**: Sections from the liver show presence of small irregular clusters of metastatic deposits in the hepatocytes and around the sinusoids (Figure 3a) whilst sections from the lung show normal architecture with presence of occasional inflammation in the alveoli (Figure 3b). The primary tumor area shows extensive areas of tissue necrosis with very minimal residual tumor cells that exhibit pleomorphism (Figure 3c). **DC+TL group**: Sections from the liver show extensive necrosis of the hepatic parenchyma, including the tumor cells (Figure 3a). The lungs showed normal architecture with presence of occasional inflammation in the alveoli (Figure 3b). The primary tumor area has undergone near total necrosis with very minimal residual tumor cells (Figure 3c). **DC+TL+TRF**: The liver tissues showed normal architecture (Figure 3a). The lungs showed mostly normal architecture with presence of occasional inflammation in the alveoli (Figure 3b). The primary tumor area appears to have undergone near total necrosis with very minimal residual tumor cells (Figure 3c). These observations suggest that the approach of using DC+TL+TRF had maximum therapeutic effects.

**Table 2:** Kruskal-Wallis Test for global comparison of lung and liver metastases amongst groups.

**Group**	**No. of Mouse**	**Primary Breast tumor differentiation**	**Lung metastasis**	**Liver metastasis**
**Untreated group**	1	Poorly differentiated	+	+
	2	Poorly differentiated	+	+
	3	Poorly differentiated	+	+
	4	Moderately differentiated	-	+
	5	Poorly differentiated	.	-
	6	Moderately differentiated	-	-
**Vehicle group**	1	Poorly differentiated	+	+
	2	Poorly differentiated	-	+
	3	Poorly differentiated	+	+
	4	Poorly differentiated	.	+
	5	Poorly differentiated	+	-
	6	Poorly differentiated	.	-
**DC group**	1	Moderately differentiated	-	-
	2	Mod differentiated	+	-
	3	Poorly differentiated	-	+
	4	Moderately differentiated	-	-
	5	Poorly differentiated	+	+
	6	Poorly differentiated	-	+
**DC+TL group**	1	Mainly necrosis tissue	-	-
	2	Necrosis	-	-
	3	Moderately differentiated with necrosis	+	+
	4	Moderately differentiated with necrosis	-	-
	5	Necrosis	-	-
	6	Moderately differentiated with necrosis	-	-
**DC+TL+TRF group**	1	Mainly necrosis tissue	-	-
	2	No tumor	-	-
	3	No tumor	-	-
	4	No tumor	-	-
	5	No tumor	-	-
	6	Mainly necrosis tissue	+	-

The *p*-value is less than 0.005 (*p*<0.05) for the lungs and liver. The metastatic deposits present in these organs were noted as present (+) and absent (-) while absence of data was indicated by (.)

### Effect of TRF on the expression of CD40 and CD80 in DC

**Figure 4:**
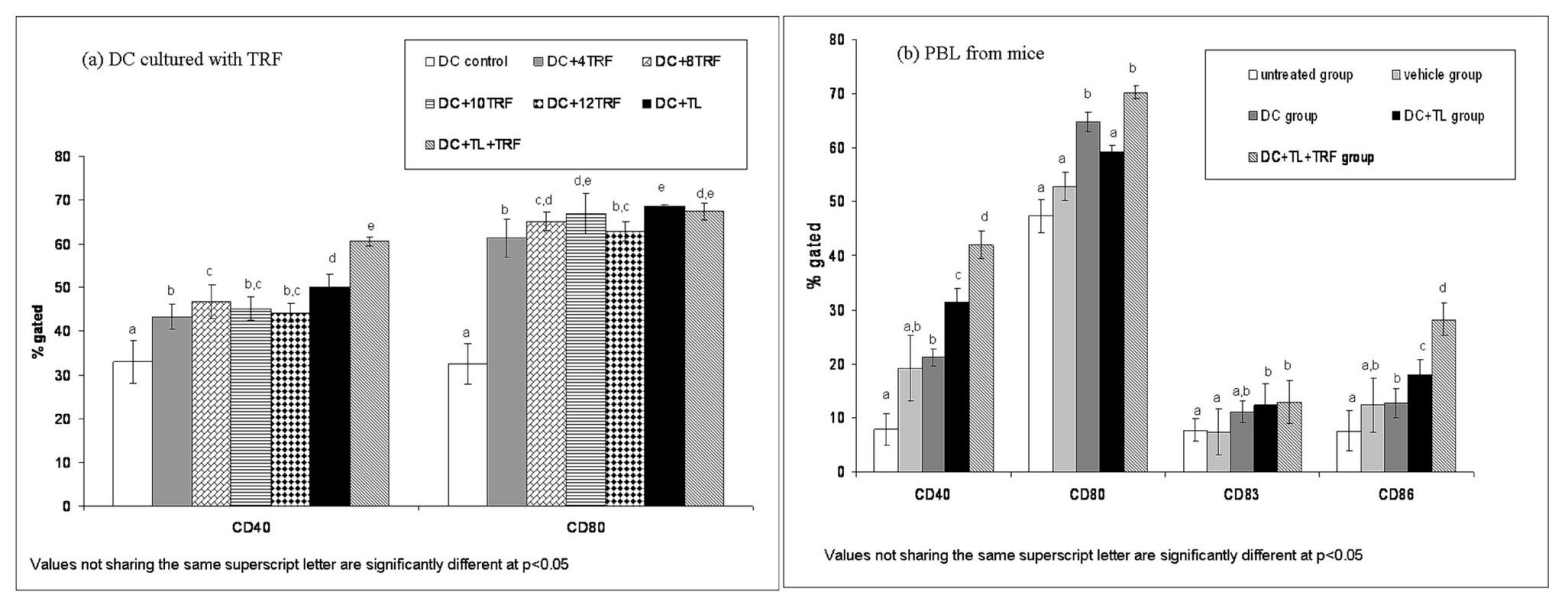
Analyzing the expression (mean value + SEM) of cell surface molecules by flow-cytometry in (a) dendritic cells harvested from naïve mice co-cultured for 72 hours with varying concentrations of TRF in the presence and absence of tumor lysate (TL), and (b) peripheral blood leucocytes (PBL) isolated from tumor-induced animals. [**Untreated**: induced with tumor; **Vehicle**: induced with tumor and fed with Soy oil; **DC**: induced with tumor and treated with DC; **DC+TL**: induced with tumor and treated with DC+TL; **DC+TL+TRF**: induced with tumor and treated with DC+TL and supplemented daily with TRF].

As shown in Figure 4a, there was an enhanced expression of CD40 and CD80 in the tumor lysate (TL)-pulsed DC that was co-cultured with TRF as compared to DC alone. However, there was a higher percentage of DC exposed to TRF (4, 8, 10 and 12 µg/mL) that expressed these molecules compared to control. There was also an apparent dose-dependent increase in the expression of CD40 and CD80 in the unpulsed DC treated with TRF, albeit to a lower extent.

### Expression of CD40, CD80, CD83 and CD86 in peripheral blood leucocytes of mice

Blood was obtained from the mice at autopsy. The peripheral blood leucocytes (PBL) were stained with specific antibodies to various cell surface markers (CD40, CD80, CD83 and CD 86) for flow cytometric analysis. As shown in Figure 4b, there lower number of cells expressing CD40, CD80, CD83 and CD86 were observed in the leucocytes from the mice in the untreated and vehicle groups compared to DC, DC+TL and DC+TL+TRF. There was a significant (p<0.05) increase in the number of leucocytes that expressed these molecules in the PBL from the DC+TL+TRF group compared to the other groups. The percentage of cells expressing CD40 increased (p<0.05) in the DC+TL+TRF group (42 + 5.26%) compared to DC+TL (31.39 + 7.2%), DC (21.16 + 4.1%), vehicle (9.18 + 5.1%) and untreated (7.9 + 3.9%) groups. Similar pattern also observed in the percentage of cells expressing CD80 and CD86 in the DC+TL+TRF group (70.2 + 17.3%; 28.2 + 9.05%) compared to others.

### Production of IFN-gamma and IL-12 by tumor-specific splenocytes

**Figure 5:**
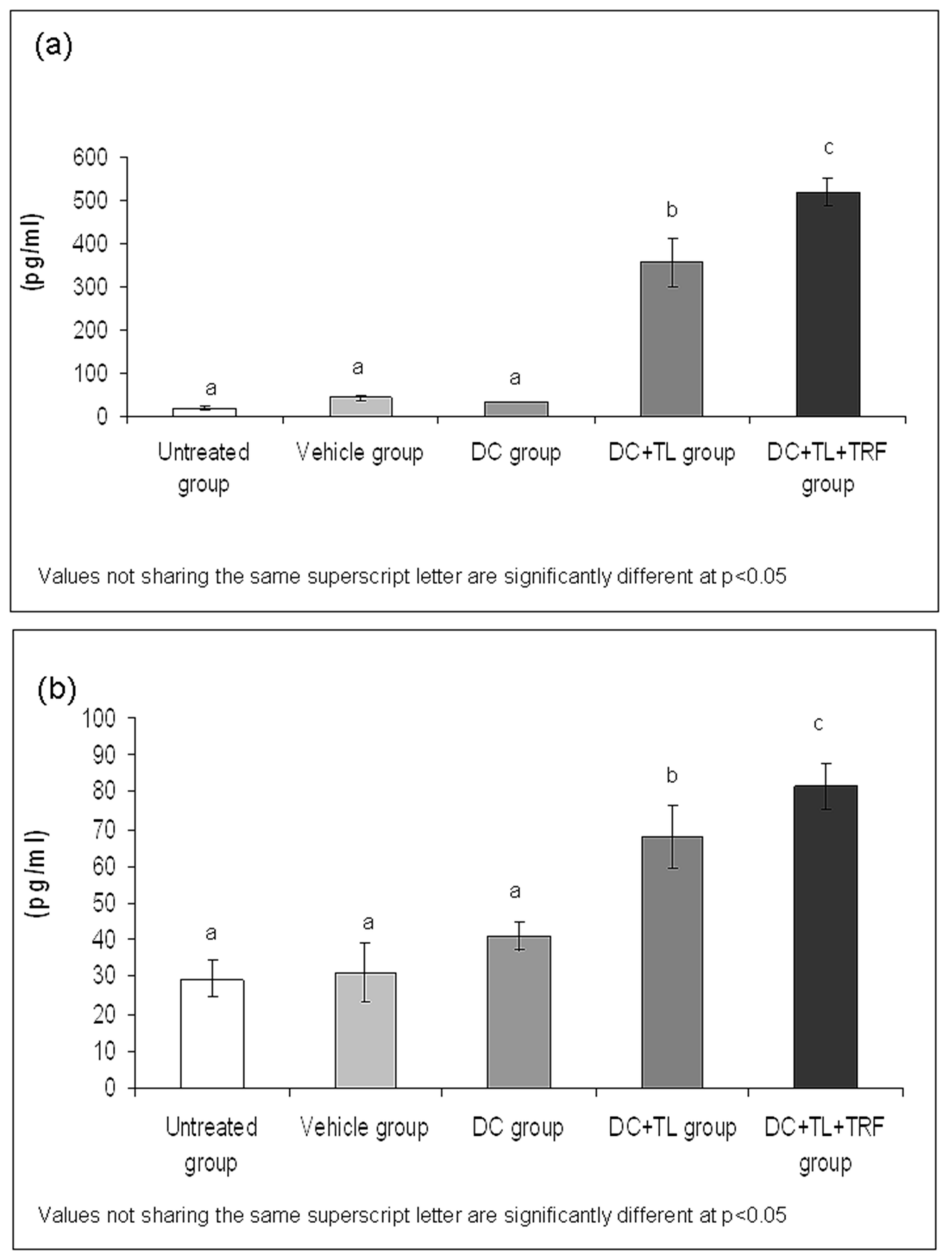
Production of (a) interferon-gamma and (b) interleukin-12 from splenocytes(mean value + SEM) harvested from the experimental mice at autopsy and co-cultured with MMC-treated 4T1 cells for 72 hours. [**Untreated**: induced with tumor; **Vehicle**: induced with tumor and fed with Soy oil; **DC**: induced with tumor and treated with DC; **DC+TL**: induced with tumor and treated with DC+TL; **DC+TL+TRF**: induced with tumor and treated with DC+TL and supplemented daily with TRF].

As shown in Figure 5, co-culture of splenocytes from the DC+TL+TRF group with MMC-treated 4T1 cells for 72 hours produced markedly (p<0.05) higher amounts of IFN-γ (517.45 + 32.26 pg/ml) as compared to splenocytes from the DC+TL group (355.69 + 56.22 pg/ml). The production of IFN-γ from splenocytes from the untreated, DC and vehicle groups was found to be 17.7 + 3.56 pg/ml, 31.92 + 0.56 pg/ml and 41.87 + 5.92 pg/ml respectively. A similar trend was observed with the IL-12 production where the highest was from the DC+TL+TRF group (81.54 + 6.19 pg/ml) followed by DC+TL group (68.14 + 8.52 pg/ml), DC group (41.17 + 3.82 pg/ml), vehicle group (31.2 + 7.91 pg/ml) and untreated group (29.5 + 4.98 pg/ml).

### CTL assay

**Figure 6:**
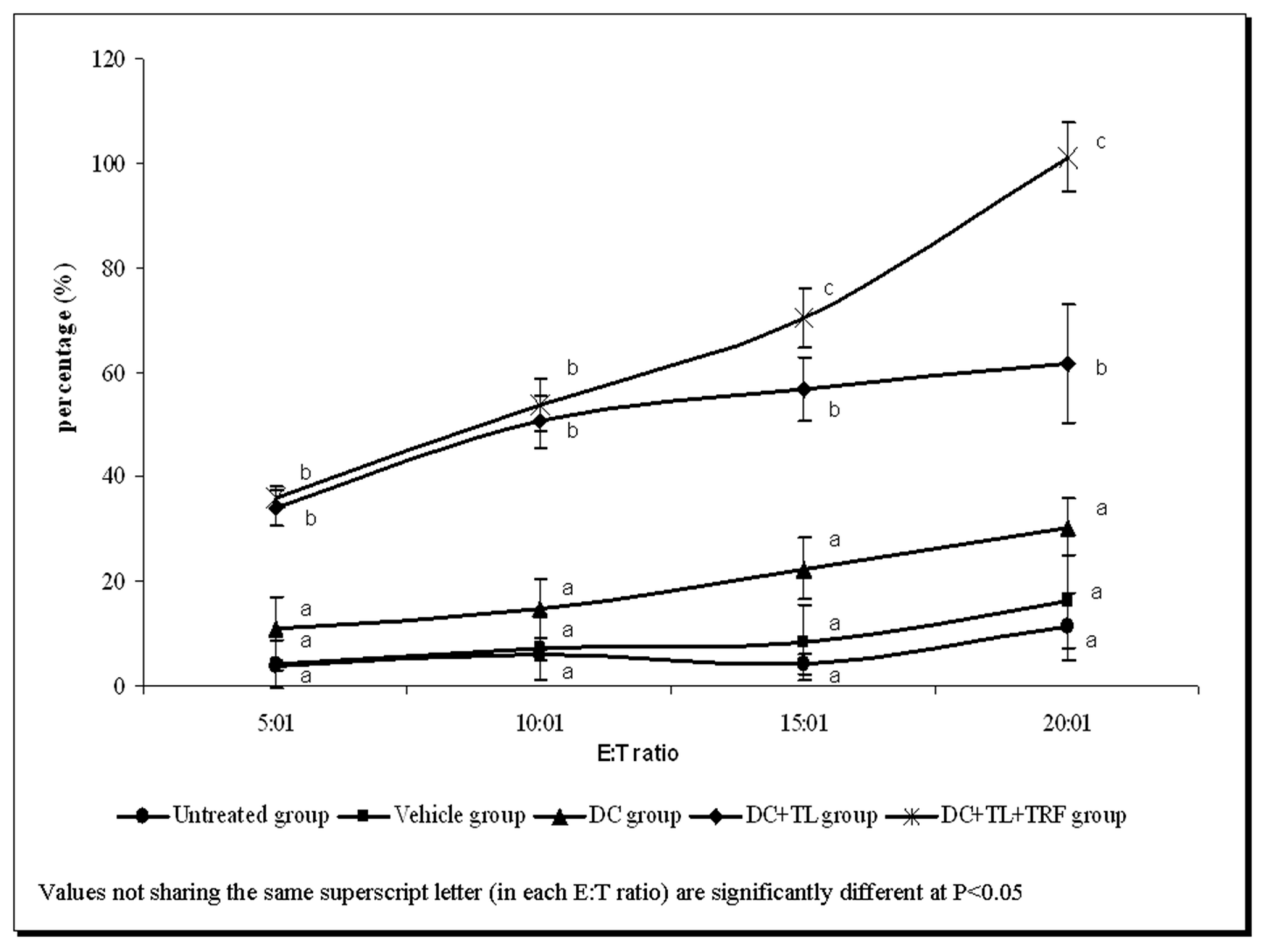
Tumor-specific cytotoxic response (mean value + SEM) by CD8^+^ T-lymphocytes isolated from the spleen of experimental mice co-cultured with MMC-treated 4T1 cells for 10 hours. [**Untreated**: induced with tumor; **Vehicle**: induced with tumor and fed with Soy oil; **DC**: induced with tumor and treated with DC; **DC+TL**: induced with tumor and treated with DC+TL; **DC+TL+TRF**: induced with tumor and treated with DC+TL and supplemented daily with TRF].

The CTL activity of CD8^+^T-cells isolated from splenocytes of mice from the untreated, DC-alone, DC+TL and DC+TL+TRF groups were compared. As shown in Figure 6, the CD8^+^ cells from the DC+TL+TRF had significantly (p<0.05) higher CTL activity compared to the DC+TL group. The CTL activity in DC+TL+TRF observed was higher in in the 5:1 E:T ratio (34 + 2.0%) compared to the other groups (DC+TL group: 30 + 3.4%, DC group: 10 + 6.0%, Vehicle and control group : 5 + 4.5%, 5 + 0.6%). This activity kept increasing with higher E:T ratios (10:1 = 56 + 4.8%; 15:1=71 + 5.7% and 20:1=99 + 6.7%). Only low levels of CTL activity were observed in untreated and vehicle groups even at higher E:T ratios. The CTL activity from the DC alone group was found to be slightly higher than the untreated and vehicle groups for all E:T ratios. These results suggested that CTL activity induced by treatment of DC+TL+TRF appeared to be specific for 4T1 tumor cells only.

## Discussion

Dendritic cells-based cancer immunotherapy is rapidly becoming a key research area in active immunotherapy [1,4-8]. It has been previously reported that pre-treatment with oral TRF supplementation for four-weeks prior to the induction of tumor has resulted in reduced tumor growth and size [25]. We have previously reported that combined approach of vaccinating mice with DC-pulsed with tumor lysate from the 4T1 cells and daily supplementation with TRF prior to the induction of tumor markedly inhibited tumor growth and produced tumor-specific response [11]. The findings from the previous study is important but has limited clinical implications as cancer patients usually seek medical help long after the cancer has been established in the patient. Hence, in the present study, we induced breast cancer in the mice first and then evaluated the efficacy of combination therapy using DC-pulsed with tumor lysate and daily TRF supplementation.

Our data show that there was minimal tumor growth in the mice that received three injections of DC-pulsed with tumor lysate and daily TRF supplementation (DC+TL+TRF). A similar enhancement of DC-based cancer vaccine has been demonstrated using a-tocopherol as an adjuvant in a murine model of lung carcinoma [6] and breast cancer [10]. The marked reduction of tumor growth observed in the mice from the DC+TL+TRF group was also corroborated with the histopathological findings where there were no signs of metastasis in the liver or lungs of these animals. There was also a marked reduction tumor growth in mice that received DC+TL or DC alone when compared to the untreated or vehicle group. Again, we show that the histopathological findings correspond with the tumor size data.

The mice in the untreated and vehicle only groups showed the highest tumor growth and extent of metastasis. In the mice from the DC alone group, there was a significant reduction in tumor growth when compared to the untreated mice but had minimal effect on metastasis. There was a marked improvement in terms of tumor growth and metastasis in mice treated with DC+TL when compared to mice that were treated with DC alone. In addition, in the DC+TL mice, there were clear signs of tumor necrosis, suggesting that destruction of tumor cells in the primary site is happening but the effect on metastasis was found to be minimal. This means that DC alone treatment had very minimal effect, whilst DC+TL treatment showed mild to moderate effect on reducing the primary tumor size. So, the best therapeutic effect was observed in the DC+TL+TRF group, as this treatment showed marked reduction in tumor size and no detectable signs of metastasis. In this study, TRF in combination DC pulsed tumor lysate treatment (DC+TL+TRF group) showed significant reduction or inhibition of tumor growth as most of the mice in this group did not develop tumors and the histopathological report, which showed mainly necrosis in tumor tissues (Table 2).

The cell surface molecules CD40 and CD80 are responsible for DC maturation and activation and also necessary for activation and survival of T-cells [25-28]. Our study shows that TRF increased the expression of CD40 and CD80 in the DC-pulsed with tumor lysate in a dose-dependent manner. The increase in expression of these cell surface molecules suggests that TRF treatment facilitate the maturation and activation of DC, thus improving the antigen presenting ability.

The splenocytes from the DC+TL+TRF animals showed higher production of IFN-γ and IL-12, which are cytokines that promote cell-mediated responses [3,29] such as tumor-specific CTL activities. Interferon-gamma [27,29,30] and IL-12 promote development of T-helper-1 (TH1) immune responses, which play a key role in producing anti-tumor immune responses. Interleukin-12 is known as a T-cell stimulating factor, which is produced by DC and macrophages in response to antigenic stimulation [31,32,33]. This cytokine can also stimulate production of IFN-γ and TNF-α by T-lymphocytes and NK cells [3,19,32,33], which enhances the cytotoxic activity of CTL [34,35].

There was a significant increase in the CTL activities observed when CD8+ T-lymphocytes isolated from the DC+TL+TRF mice were cultured with MMC-treated 4T1 cells. The CTL activity observed was found to be tumor-specific. The PBL from the DC+TL+TRF mice also showed up-regulation CD40, CD80, CD83 and CD86, which suggests that TRF supplementation may have facilitated the antigen presentation ability of the DC in the PBL by promoting their maturation and activation *in-vivo*. It has been reported that mature DC are able to migrate more rapidly to secondary lymphoid organs, where they can rapidly initiate anti-tumor T-cell responses [2,34]. Taken as a whole, our data shows that combine treatment using tumor lysate pulsed DC (DC+TL) and TRF supplementation as an adjuvant was efficacious in inhibiting tumor growth and also in promoting tumor-specific immune responses.

## Conclusion

In this study, we report for the first time that TRF-adjuvanted DC immunotherapy (DC+TL) can suppress the growth of breast cancer in mice with palpable tumors. This treatment approach showed marked reduction in tumor growth and metastasis. We would like to proposed that oral supplementation with TRF can be used as an adjuvant to selectively kill tumor cells and also to enhance the tumor–specific immune responses. This concept of using TRF supplementation together with treatment with tumor lysate-pulsed DC (DC+TL) to enhance anti-tumor responses is novel and needs further study in experimental and clinical settings.
